# Study on association between genetic polymorphisms of haem oxygenase-1, tumour necrosis factor, cadmium exposure and malaria pathogenicity and severity

**DOI:** 10.1186/1475-2875-9-260

**Published:** 2010-09-17

**Authors:** Jiraporn Kuesap, Kenji Hirayama, Mihoko Kikuchi, Ronnatrai Ruangweerayut, Kesara Na-Bangchang

**Affiliations:** 1 Pharmacology and Toxicology Unit, Graduate Programme in Biomedical Sciences, Thammasat University, Pathumthani, Thailand; 2 Department of Molecular Immunogenetics, Institute of Tropical Medicine, Nagasaki University, Japan; 3 Mae Sot General Hospital, Tak Province, Thailand

## Abstract

**Background:**

Malaria is the most important public health problems in tropical and sub-tropical countries. Haem oxygenase (HO) enzyme and the pro-inflammatory cytokine tumour necrosis factor (TNF) have been proposed as one of the factors that may play significant role in pathogenicity/severity of malaria infection. HO is the enzyme of the microsomal haem degradation pathway that yields biliverdin, carbon monoxide, and iron. In this study, the association between malaria disease pathogenicity/severity and (GT)_n _repeat polymorphism in the promoter region of the inducible HO-1 including the effect of cadmium exposure (potent inducer of HO-1 transcription) as well as polymorphism of TNF were investigated.

**Methods:**

Blood samples were collected from 329 cases non-severe malaria with acute uncomplicated *Plasmodium falciparum *malaria (UM) and 80 cases with *Plasmodium vivax *malaria (VM), and 77 cases with severe or cerebral malaria (SM) for analysis of genetic polymorphisms of HO-1 and TNF and cadmium levels. These patients consisted of 123 (25.3%) Thai, 243 (50.0%) Burmese and 120 (24.7%) Karen who were present at Mae Sot General Hospital, Mae Sot, Tak Province, Thailand.

**Results:**

The number of (GT)_n _repeats of the HO-1 gene in all patients varied between 16 and 39 and categorized to short (S), medium (M) and long (L) GT_n _repeats. The genotype of (GT)_n _repeat of HO-1 was found to be significantly different among the three ethnic groups of patients. Significantly higher frequency of S/L genotype was found in Burmese compared with Thai patients, while significantly lower frequencies of S/S and M/L but higher frequency of M/M genotype was observed in Burmese compared with Karen patients. No significant association between HO-1 and TNF polymorphisms including the inducing effect of cadmium and malaria pathogenicity/severity was observed.

**Conclusions:**

Difference in the expression of HO-1 genotype in different ethnic groups may contribute to different severity of malaria disease. With this limited sample size, the finding of the lack of association between malaria disease pathogenicity/severity genetic polymorphisms of HO-1 (GT)_n _repeat as well as TNF observed in this study may not entirely exclude their possible link with malaria disease pathogenicity/severity. Further study in larger sample size is required.

## Background

Malaria remains an important health problem in several countries in the world especially in Thailand [1,2]. Cerebral malaria is one of the major pathological complications of *Plasmodium falciparum *infection in humans, manifesting as coma that can lead to death. The pathogenesis of cerebral malaria remains controversial, but major factors involved, *i.e*., cytokines and adhesion molecules are well documented [3]. In Thailand, malaria is still problematic in particular areas, especially in the forest and forest fringes along international borders where there is significant population movement. The highest cases of malaria in 2009 were reported from Tak Province, where Mae Sot District is located. They have carried the largest burden of malaria diagnosis and treatment for foreigners, the majority of whom are from Myanmar [2]. Mae Sot District is also an endemic area polluted with high levels of cadmium [4-6]. The degree of soil cadmium contamination is associated with rice grain cadmium contamination downstream of an actively mined zone of zinc mineralization in Mae Sot District [6]. Population cadmium exposure is due mainly to eating contaminated food, smoking cigarettes, and occupation in cadmium-contaminated places [7]. There have been several reports on the involvement of cadmium in nephropathy [5,7,8].

Haem oxygenase (HO) enzyme is proposed as one of the factors that may play role in pathogenesis including susceptibility and severity of malaria disease [9,10]. The isoforms HO-1 and HO-2, are microsomal enzymes that play important role in haem catabolism to produce biliverdin/bilirubin, carbonmonoxide and iron [9,11-13]. The promoter region of HO-1 gene contains cadmium response element (CdRE), the binding site for cadmium which is a potent inducer of HO-1 transcription [14]. Therefore, it may be possible that the linkage of cadmium exposure and susceptibility to malaria disease is through binding of cadmium to CdRE of the HO-1 gene. The polymorphism of human HO-1 gene promoter compose of the single nucleotide polymorphism (SNPs) and (GT)_n _dinucleotide polymorphism which may contribute to the fine tuning of the transcription. Long (GT)_n _alleles have been found associated with susceptibility to smoking-induced emphysema or coronary artery disease, while they may be linked to resistance to cerebral malaria [10,15,16].

TNF is a cytokine produced primarily by monocytes and macrophages in many inflammatory diseases. SNPs of the TNF promoter have been reported to be associated with susceptibility to severe malaria. Four mutations are transitions of adenosine to guanine and are located at nucleotide positions -238, -244, -308, and -376, relative to the transcriptional site [17,18]. Various studies have investigated the relation existing between TNF promoter alleles and severity of malaria. TNF has been shown to be elevated in severe malaria [19], and TNF promoter region -238A allele together with -376A allele are associated with susceptibility to severe malaria [20,21]. Furthermore, TNF P-D allele has been shown significantly associated with cerebral malaria in our previous study in Karen and Burmese populations [22].

In this study, the (GT)_n _repeat polymorphism in the promoter region of the inducible HO-1 and six mutations of TNF from malaria patients (Thai, Burmese and Karen), in Mae Sot endemic area, were analysed. The aim was to determine the association between genetic polymorphisms of HO-1, TNF and severity of malaria infection.

## Methods

### Subjects and blood collection

A total of 486 malaria patients infected with *P. falciparum *and *P. vivax *(aged between three and 73 years, parasitaemia ranged from 77 and 1,840,000/μl) comprising 123 (25.3%) Thai, 243 (50.0%) Burmese and 120 (24.7%) Karen who were present at Mae Sot General Hospital, Mae Sot, Tak Province, Thailand, were recruited into the study. The study protocol was approved by the Ethics Committee of the Ministry of Public Health, Thailand. All patients gave informed consents for study participation prior to the study enrolment.

Blood samples were collected from 329 cases non-severe malaria with acute uncomplicated *P. falciparum *malaria (UM) and 80 cases with *P. vivax *malaria (VM), and 77 cases with severe or cerebral malaria (SM) for analysis of genetic polymorphisms of HO-1 and TNF and cadmium levels.

### Extraction of genomic DNA

Genomic DNA was extracted from packed white blood cells (buffy coat layers) using standard phenol-chloroform extraction technique [23].

### Microsatellite polymorphism

To amplify the (GT)_n _microsatellite located at position -270 of HO-1 gene promoter, the PCR assays were performed over 35 cycles of 30 s at 95°C, 30 s at 60°C, and 3 min at 72°C. A fluorescently labelled primer p1 (5'-AGAGCCTGCAGCTTCTCAGA-3') and an unlabeled antisense primer p2 (5'-ACAAAGTCTGGCCATAGGAC-3') was designed according to the published sequence [24]. The PCR products were analysed by a laser-based automated DNA sequencer, ABI 3730 DNA Analyzer (Applied Biosystems, Foster City, Calif., USA).

### Direct sequencing

To analysis of six-biallelic polymorphic sites (TNF-1,031T/C, -863C/A,-857C/T, -376G/A, -308G/A, and -238G/A) in the 5'-flanking region of TNF gene, a 1,042-bp DNA fragment spanning the 5'-flanking region of the TNF gene from position -66 to -1,107 were amplified by PCR. The primers for the PCR reactions and the method for direct sequencing followed the methods described by Higuchi [25] and Ubalee [22].

### Analysis of cadmium levels

Cadmium levels in plasma samples collected from 138 patients with malaria (13 cases with SM, 92 cases with UM and 33 cases with VM) were measured by Graphite Furnace Atomic Absorption Spectrophotometer (GFAAS) [26]. Analysis of cadmium in blood was not possible due to limited blood sample volume. The threshold level of blood cadmium defined by the World Health Organization is 0.5 μg/L [27].

### Statistical analysis

Statistical analysis was performed using SPSS version 12.0 software (SPSS Co., Ltd., Thailand). Statistical significance level for all tests was set at α = 0.05. The frequencies of HO-1 and TNF genotype alleles were summarized as number of cases and percentage values. The association between genetic polymorphisms of HO-1 including TNF and ethnicity and malaria disease pathogenicity/severity (UM, SM, VM) were determined using Chi-square test. The concentrations of cadmium in plasma were presented as median (range) values for data not conforming to normal distribution. The association between the level of cadmium exposure and malaria disease pathogenicity/susceptibility/severity was performed by Kruskal Wallis tests. Difference in proportions among ethic groups and disease pathogenicity/susceptibility/severity was determined by Chi-square test.

## Results

### The polymorphism of (GT)_n _repeat of human HO-1 and association with malaria disease pathogenicity/susceptibility/severity

**Figure 1:**
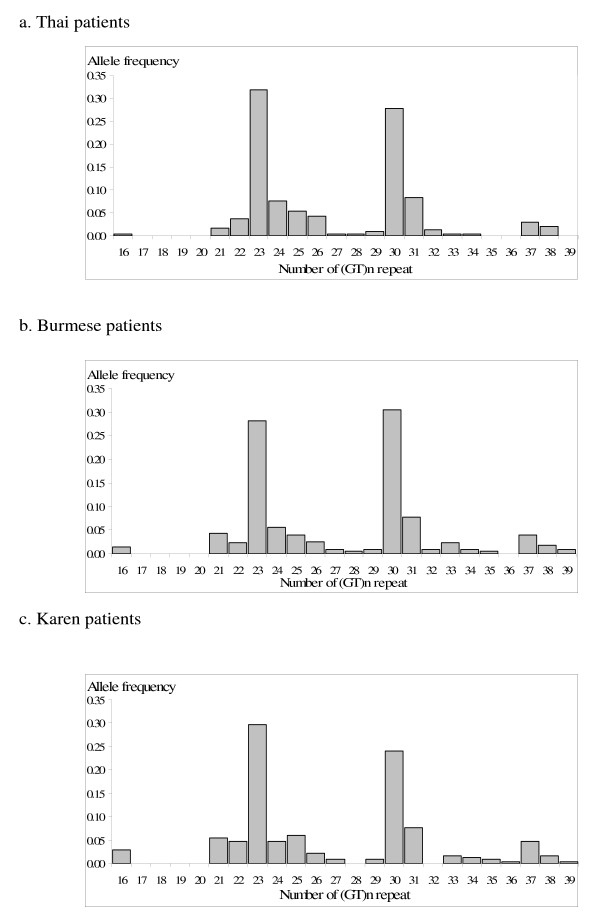
**Frequency distribution of (GT)_n _repeats in three ethnic groups of patients**.

The number of (GT)_n _repeats of the HO-1 gene varied between 16 and 39 in all patients (Figure 1). It was categorized to short (16-27), medium (28-33) and long (34-39) of GT_n _repeats. The allele frequencies of (GT)_n _allele were similar in the three ethnic groups. With regards to the frequencies of the genotypes (S/S, S/M, S/L, M/M, M/L, L/L) of (GT)_n _repeats (Table 1), significantly higher frequency of S/L genotype was found in Burmese compared with Thai patients. However, significantly lower frequencies of S/S and M/L but higher frequency of M/M genotype was found in Burmese compared with Karen patients. When data were combined for all ethnic groups, no association between malaria disease severity and HO-1 genotype was observed.

**Table 1:** Distribution of HO-1 promoter genotypes and allele frequencies of the three ethnics (Thai, Burmese, Karen) of malaria patients

	**Ethnic**		
	**Thai**	**Burmese**	**Karen**
	
*n*	119	240	118
Alleles, *n *(%)			
S	132 (55.5%)	236 (49.2%)	133 (56.4%)
M	93 (39.1%)	205 (42.7%)	81 (34.3%)
L	13 (5.5%)	39 (8.1%)	22 (9.3%)
			
Genotypes, *n *(%)			
S/S	39 (32.8%)	57 (23.8%)^a^	40 (33.9%)
S/M	50 (42.0%)	97 (40.4%)	45 (38.1%)
S/L	4 (3.4%)^b^	25 (10.4%)	8 (6.8%)
M/M	18 (15.1%)	49 (20.4%)^c^	12 (10.2%)
M/L	7 (5.9%)	10 (4.2%)^d^	12 (10.2%)
L/L	1 (0.8%)	2 (0.8%)	1 (0.8%)

^a ^*p *= 0.042, 95% CI of odd ratio: 0.37-0.98 for Burmese *vs *Karen (Chi-square test)

^b ^*p *= 0.021, 95% CI of odd ratio: 0.10-0.88 for Thai *vs *Burmese (Chi-square test)

^c ^*p *= 0.015, 95% CI of odd ratio: 1.15-4.45 for Burmese *vs *Karen (Chi-square test)

^d ^*p *= 0.026, 95% CI of odd ratio: 0.16-0.92 for Burmese *vs *Karen (Chi-square test)

### The polymorphism of TNF alleles and association with malaria pathogenicity/susceptibility/severity

The patterns of SNPs in TNF alleles in the three ethnic groups were observed based on five [22] and three [28] point mutations as shown in Tables 2 and 3. For the five SNPs of biallelic polymorphism sites (-1031, -863, -857, -308, -238), six types of TNF promoter (TNF P) allele polymorphism were found, *i.e*., TNF P-A, TNF P-B, TNF P-C, TNF P-D, TNF P-E and TNF P-F. For the pattern of three SNPs of biallelic polymorphism sites (-1031, -863, -857), five types of TNF P allele polymorphism were observed, *i.e*., TNF P-1, TNF P-2, TNF P-3, TNF P-4 and TNF P-5. There was no significant difference in the frequencies of each mutation among all the three ethnic groups. In addition, when data were combined for all ethnic groups, lack of association between any pattern of TNF allele polymorphism as well as each individual single SNP and malaria disease pathogenicity/severity was found.

**Table 2:** Six TNF alleles polymorphism (TNF P-A, TNF P-B, TNF P-C, TNF P-D, TNF P-E, TNF P-F) based on five SNPs of biallelic polymorphism sites (-238, -308, -857, -863, -1031) in patients with severe (SM), uncomplicated falciparum (UM) and vivax malaria (VM) cases when data were combined for the three ethnic groups

TNF type	Polymorphic sites of the promoter region of TNF-α gene					SM	UM	VM
				
	-238	-308	-857	-863	-1031	(%)	(%)	(%)
TNFP-A	G	G	C	C	T	41.3	52.3	53.5
TNFP-B	G	G	C	A	C	39.7	33.8	33.8
TNFP-C	A	G	C	C	C	3.2	3.3	4.2
TNFP-D	G	G	T	C	T	6.3	4.3	3.5
TNFP-E	G	A	C	C	T	7.9	5.0	3.5
TNFP-F	G	G	C	A	T	1.6	1.3	1.4

**Table 3:** Five TNF alleles polymorphism (TNF P-1, TNF P-2, TNF P-3, TNF P-4, TNF P-5) based on three SNPs of biallelic polymorphism sites (-857, -863, -1031) in patients with severe (SM), uncomplicated falciparum (UM) and vivax malaria (VM) cases when data were combined for the three ethnic groups

TNF type	Polymorphic sites of the promoter region of TNF-α gene			SM	UM	VM
				
	-857	-863	-1031	(%)	(%)	(%)
TNFP-1	C	C	T	49.2	57.2	57.7
TNFP-2	T	C	T	6.3	4.3	3.5
TNFP-3	C	A	C	39.7	34.5	34.5
TNFP-4	C	C	C	3.2	3.0	3.5
TNFP-5	C	A	T	1.6	1.0	0.7

### Cadmium levels and association with malaria pathogenicity/susceptibility/severity

When data were combined for the three ethnic groups, a trend of higher plasma cadmium levels in falciparum infection [median (range) values of 0.16 (0.03-2.34) and 0.24 (0.01-3.18) μg/L for SM and UM, respectively] compared with VM [0.10 (0.01-0.33) μg/L], was observed. In the group with cadmium levels < 0.5 mg/L, the proportions of patients with SM (n = 10, 76.9%) and UM (n = 74, 80.4%) were significantly lower than VM (n = 33, 100%), and in addition, the proportion of Thai patients (n = 18, 66.7%) was significantly lower than Burmese (n = 81, 91.0%).

## Discussion

The association between human haem oxygenase-1 (HO-1) and pathogenicity/severity of malaria infection was described in a few studies *in vitro *including molecular epidemiological investigation from brain lesion [24,29,30] and the hypothetical roles of HO-1 in pathogenesis of cerebral malaria was proposed by Shibahara [10]. However, only one research article which provides evidence on the association between (GT)_n _repeat polymorphism of HO-1 and malaria pathogenicity/severity was reported in Karen ethnic minority group with cerebral malaria and acute uncomplicated falciparum malaria who resided in areas along the Thai-Myanmar border [24]. In that study, the proportion of patients with short (GT)_n _alleles was found to be significantly higher in cerebral malaria (SM) than UM. Short (GT)_n _alleles in the promoter region may therefore represent a genetic risk factor for cerebral malaria as in may directly enhance the transcription of HO-1 in malaria patients, and as a consequence, the products of haem degradation, *i.e*., CO, iron and bilirubin, may increase in the brain lesion. Furthermore, the influence of the (GT)_n _repeat polymorphisms in the HO-1 gene promoter was investigated in different cell lines where constructs with lengths of less than 25 repeats (short repeat) showed an increasing HO-1 basal promoter activity [31] or transcriptional up-regulation in response to various stimuli as compared to that with greater than 25 repeats [15]. Increasing transcriptional up-regulation leads to high activity of HO-1 enzyme and thus, the haem degradation products particularly iron, which is an essential supply for growth and proliferation of malaria parasite. Excess amount of iron might trigger high parasitaemia causing red blood cell haemolysis and severe malaria including cerebral malaria.

The present study, due to limitation of available data on (GT)_n _repeat polymorphism of HO-1 in different patient populations with different malaria severity, estimation of proper sample size was not possible. There appears to be a significant difference in HO-1 genotypes among the three ethnics. This may explain difference in pathogenicity/severity of malaria infection in various ethnics. The frequencies of short (GT)_n _alleles (S/S) genotype in the three groups of patients with different disease severity (combined ethnics) were found to be comparable. This lack of association could represent a genuine finding, or else, a low study power due to inadequate sample size (particularly in the SM group). In addition, the major confounding factor ethnics cannot be excluded. In a previous study, a significant association between the short (GT)_n _alleles (S/S) polymorphism of HO-1 gene promoter and increased risk of cerebral malaria (severe malaria) has been demonstrated in 150 Burmese and Karen patients [24]. Nevertheless, the sample size included in that study was unbalanced and also too small (30 cases of cerebral malaria, 120 cases of UM) to provide a definitive conclusion on such association. The finding of the lack of association between genetic polymorphism of HO-1 (GT)_n _repeat and malaria disease pathogenicity/severity found in this study may not entirely exclude the possible link between HO-1 enzyme activity and malaria disease pathogenicity/severity. Results from recent *in vitro *study provide insight into the molecular mechanism of induction of HO-1 activity and the link with malaria pathogenicity/severity [10,24]. Treatment of astrocyte (CCF-STTG1) and retinal pigment epithelial (ARPE-19, D407) cells with exogenous prostaglandin D_2 _(PGD_2_) and 15d-PGJ_2 _(metabolite of PGD_2_) consistently induced the expression of HO-1 mRNA and protein [32,33]. PGD_2 _is a major prostanoid produced in the brain and is involved in the regulation of sleep and pain responses [34].

Cadmium has been shown to be a potent inducer of HO-1 activity [14,35], and as a consequence, this might be linked to severity of malaria disease. Blood cadmium reflects recent cadmium exposure, whereas urinary cadmium indicates body accumulation of cadmium [7,36]. The ratio of plasma to blood cadmium varies from 3 to 16% [37]. The observed median (range) values of plasma cadmium concentrations in the VM, UM and SM groups of patients were 0.10 (0.01-0.33), 0.24 (0.01-3.18) and 0.16 (0.03-2.34) μg/L, respectively. Twenty-one patients (in SM and UM groups) had the concentrations above the tolerance limit of 0.5 μg/L [38]. The information on blood cadmium concentrations in Thai population has been reported in non-occupational females living in Bangkok and other areas, with the geometric mean blood levels of 0.40 and 0.43 μg/L, respectively [39]. The observed concentrations of cadmium in plasma in malaria patients were considered higher than non-occupational residents in other areas due to the fact that cadmium level in plasma is only about 3-16% of that in blood [40]. Although the concentrations of plasma cadmium are still considered low considering the threshold level of 0.5 mg/L, results may more or less explain the high incidence of malaria case report, mortality and morbidity from this high cadmium pollutant area of Mae Sot. It appears that plasma cadmium concentrations tended to be higher in patients with falciparum malaria (UM and SM) compared with VM. In addition, significantly lower proportions of cases with falciparum malaria including both UM (80.4%) and SM (76.9%) were found compared with VM (100%) at plasma cadmium level of ≤ 0.5 μg/L. Cadmium is known to produce toxic effect to human and other organisms including malaria parasite when exposing with high level of cadmium. It is possible that optimally high levels of cadmium might promote the growth of *P. falciparum *but not with *P. vivax *by inducing HO-1 activity to produce iron. The mechanism(s) by which parasite transport or demolish cadmium are, therefore, important for their survival. It has been reported that *P. falciparum *efflux cadmium from cells through PFMDR2 protein [41]. Again, due to limited sample size and confounding factors (age, gender, ethnic), definitive conclusion on this association could not be obtained. Cadmium concentrations have been reported to vary markedly according to age, gender and ethnic [42,43] and results from the present study also revealed some association between ethnicity (Thai *vs *Burmese) and cadmium levels in group with level of ≤ 0.5 μg/L.

Several articles have reported the involvement of cytokines in the histopathology of cerebral malaria [44,45]. Among these cytokines, TNF is thought to play a major role in pathogenesis of cerebral malaria. Others have reported the presence of cytokines in the brain after fatal cases of malaria [45,46]. Three SNPs of TNF promoter -857, -863 and -1031 were analyzed and the frequency of the designated allele -857C/-863C/-1031C was found to be significantly higher in patients with cerebral malaria than that with UM in area along Thai-Myanmar border [28]. In the present study, the frequency of -857C/-863C/-1031C was found to be 1.3, 4.0 and 3.1% in Thai, Burmese and Karen patients, respectively. It was noted however for the observed lack of significant difference in frequency of this promoter allele (-857C/-863C/-1031C) between the SM and UM groups. The TNF polymorphism was categorized based on the patterns of allele polymorphisms reported by Ubalee [22] and Hananantachai [28]. The first classification-- TNF P-A, TNF P-B, TNF P-C, TNF P-D, TNF P-E and TNF P-F was based on the biallelic polymorphism sites at nucleotides -238, -308, -857, -863 and -1031. The second classification-- TNF P1, TNF P-2, TNF P-3, TNF P-4 and TNF P-5 was based on the biallelic polymorphism sites at nucleotides -857, -863 and -1031. No significant association between any pattern of TNF polymorphism as well as each individual single SNPs and disease pathogenicity/severity (SM, UM, VM) including ethnics (Thai, Karen, Burmese) were observed when polymorphisms were classified based on both criteria. The biallelic polymorphic sites at nucleotides -238, -308, -857, -863 and -1031, with seven allele patterns (TNF P-A, TNF P-B, TNF P-C, TNF P-D, TNF P-M1, TNF P-M4 and TNF P-M7) were identified in a previous study in Burmese and Karen patients who lived near Thai-Myanmar border [22] and TNF P-D allele was found to be significantly associated with the high proportion of cerebral malaria (SM) compared with UM cases. Interestingly, the TNF P-M1, TNF P-M4 and TNF P-M7 but TNF P-E and TNF P-F were not found in all the three ethnics under the present investigation. The frequencies of the -863C or -1031T allele, the typical polymorphisms of TNF P-D, were found much higher in Swedish (94% for -863C and 80% for -1031T) [47] and in Japanese (82% for -863C and 84% for -1031T) subjects [25] than in the three ethnics under present investigation where the frequencies of -863C and -1031T alleles were 38 *vs *39% in Thai, 40 *vs *38% in Burmese and 41 *vs *40% in Karen patients, respectively. The association of single SNP of TNF was also found in other studies in African patients. Two studies were performed in Gambian children and results showed that the TNF -238 A allele was found to be associated with susceptibility to severe malarial anaemia but not to CM [20]. On the other hand, the neighbouring TNF -308 A allele was associated with CM but not with severe malarial anaemia [48]. Furthermore, data from 1,048 Kenyan children showed the TNF -308 A allele to be significantly associated with high density *P. falciparum *parasitaemia. Among low birth weight children, the TNF -308 A allele was also found to be associated with severe anaemia with a trend toward a risk for severe malaria anaemia [49]. In contrast with other studies, a study conducted in Gabon comparing malaria severity and TNF promoter (TNF P) variants has demonstrated that the frequencies of carriers of distinct TNF variants did not differ significantly in the groups with mild and SM [18]. Moreover, TNF plasma levels were not significantly associated with any of the TNF variants.

## Conclusions

Difference in the expression of HO-1 genotype in different ethnic groups may contribute to different severity of malaria disease. To definitely conclude on the association between malaria pathogenicity/severity and the involvement of genetic polymorphisms of HO-1 (including the inducing effect of cadmum) and TNF, further study with larger sample size estimation based on this preliminary data should be performed with focus on only one ethnic group (Burmese patients, which constitute the majority group of patients in this area).

## Competing interests

The authors declare that they have no competing interests.

## Authors' contributions

JK performed the molecular genetic studies, participated in the sequence alignment, interpretation of data, performed the statistical analysis and drafted the manuscript. KH participated in the design of the study. MK participated in the molecular genetic studies and sequence alignment. RR participated in sample collection and the study design and coordination. KN participated in the design of the study, performed the statistical analysis and finalised the manuscript. All authors read and approved the final manuscript.
